# Comparative transmission of SARS-CoV-2 Omicron (B.1.1.529) and Delta (B.1.617.2) variants and the impact of vaccination: national cohort study, England

**DOI:** 10.1017/S0950268823000420

**Published:** 2023-03-20

**Authors:** Hester Allen, Elise Tessier, Charlie Turner, Charlotte Anderson, Paula Blomquist, David Simons, Alessandra Løchen, Christopher I. Jarvis, Natalie Groves, Fernando Capelastegui, Joe Flannagan, Asad Zaidi, Cong Chen, Christopher Rawlinson, Gareth J. Hughes, Dimple Chudasama, Sophie Nash, Simon Thelwall, Jamie Lopez-Bernal, Gavin Dabrera, André Charlett, Meaghan Kall, Theresa Lamagni

**Affiliations:** 1 COVID-19 Vaccines and Epidemiology Division, UK Health Security Agency, Colindale, 61 Colindale Avenue, London NW9 5EQ, UK; 2 Field Service, UK Health Security Agency, London NW9 5EQ, UK; 3 Genomics Cell, UK Health Security Agency, London NW9 5EQ, UK; 4 Immunisation and Countermeasures Division, UK Health Security Agency, 61 Colindale Avenue, London NW9 5EQ, UK; 5 Statistics, Modelling and Economics Division, UK Health Security Agency, 61 Colindale Avenue, London NW9 5EQ, UK; 6 Joint Modelling Team, UK Health Security Agency, Porton Down, Salisbury, Wiltshire, SP4 0JG, UK

**Keywords:** Contact tracing, England, epidemics, Omicron, population surveillance, SARS-CoV-2, transmissibility, vaccination

## Abstract

The severe acute respiratory syndrome coronavirus 2 (SARS-CoV-2) Omicron variant (B.1.1.529) rapidly replaced Delta (B.1.617.2) to become dominant in England. Our study assessed differences in transmission between Omicron and Delta using two independent data sources and methods. Omicron and Delta cases were identified through genomic sequencing, genotyping and S-gene target failure in England from 5–11 December 2021. Secondary attack rates for named contacts were calculated in household and non-household settings using contact tracing data, while household clustering was identified using national surveillance data. Logistic regression models were applied to control for factors associated with transmission for both methods. For contact tracing data, higher secondary attack rates for Omicron *vs.* Delta were identified in households (15.0% *vs.* 10.8%) and non-households (8.2% *vs.* 3.7%). For both variants, in household settings, onward transmission was reduced from cases and named contacts who had three doses of vaccine compared to two, but this effect was less pronounced for Omicron (adjusted risk ratio, aRR 0.78 and 0.88) than Delta (aRR 0.62 and 0.68). In non-household settings, a similar reduction was observed only in contacts who had three doses *vs.* two doses for both Delta (aRR 0.51) and Omicron (aRR 0.76). For national surveillance data, the risk of household clustering, was increased 3.5-fold for Omicron compared to Delta (aRR 3.54 (3.29–3.81)). Our study identified increased risk of onward transmission of Omicron, consistent with its successful global displacement of Delta. We identified a reduced effectiveness of vaccination in lowering risk of transmission, a likely contributor for the rapid propagation of Omicron.

## Introduction

Severe acute respiratory syndrome coronavirus 2 (SARS-CoV-2) variant B.1.1.529 was first detected on 9 November 2021 in South Africa and by 26 November classified as a ‘variant of concern’ by the World Health Organisation (WHO), with the designation ‘Omicron’ [1]. By this point, the variant had probably reached several countries.

The first Omicron case in England, confirmed by genomic sequencing, was detected on 16 November 2021, when the dominant variant circulating was Delta [2]. A dramatic rise in incidence of the coronavirus disease 2019 (COVID-19) in England ensued, reaching the highest incidence reported to date, with over 245 000 daily cases diagnosed by late December 2021. Preliminary analyses suggested Omicron was associated with increased transmission and reduced vaccine effectiveness compared to other SARS-CoV-2 variants [3]. Data from Gauteng province, South Africa indicated rapid spread of this variant with twice as many cases in their fourth wave compared to previous waves [4].

Previous emerging SARS-CoV-2 variants, Alpha and Delta, established dominance in England, with increased household clustering demonstrated for both these variants [5–7]. In contrast, Beta and Gamma variants have (to date) failed to gain a strong foothold since emergence [3, 8]. As such, assessing new variants in both household and non-household settings to understand intrinsic transmissibility and immune evasion is essential to inform decisive and rapid public health actions.

We assessed and compared the transmissibility SARS-CoV-2 Omicron variant during the period of its emergence alongside the dominant Delta variant using two different methods.

## Methods

We assessed transmission of SARS-CoV-2 from cases confirmed as Omicron or Delta using two different data sources and analytical methods:

1. *Transmission to named contacts:* Secondary attack rates and risk of transmission to named household and non-household contacts of cases using contact tracing data.
2. *Household clustering*: Risk of clusters within cases' households using national surveillance data.

To differentiate between the two analyses, we have applied different terminology. For the transmission to named contacts analysis, the primary SARS-CoV-2 case included in the analysis is referred to as the ‘exposer’ and the outcome of interest, a named contact becoming a secondary case, is referred to as ‘transmission’. For the household clustering analysis, the primary SARS-CoV-2 case is referred to as the index case and the outcome of interest, more than one case within a household, is referred to as household clustering.

### Data sources

#### National surveillance data for cases

Analyses were based on cases in England identified as Delta or Omicron with specimen dates between 05 and 11 December 2021, when both variants were circulating.

Positive SARS-CoV-2 tests in England are reported by private and National Health Service (NHS) laboratories to the UK Health Security Agency (UKHSA). Laboratory and self-reported positive rapid Lateral Flow Device (LFD) testing data are stored within the UKHSA Second Generation Surveillance System [8–10].

Genomic sequencing is co-ordinated by the COG-UK (COVID-19 Genomics UK) consortium and held in the Cloud Infrastructure for Big Data Microbial Bioinformatics database (CLIMB) [11]. SARS-CoV-2 variants were identified through genomic sequencing and genotyping and the identification of S-gene target failure (SGTF) in PCR confirmed cases. Variant identification is based on UKHSA's single and multinucleotide polymorphisms definitions [8]. Specimens are selected for sequencing through geographic-weighted population-level sampling of community cases supplemented by targeted selection including recent international travellers, care homes or NHS laboratories. Samples are eligible for sequencing or genotyping if they have a CT value threshold of ≤30CT for at least one gene target and ≤30CT for both N and ORF1ab gene for defining SGTF. The positive predictive value of SGTF for classifying Omicron was ≥99% during the study period [12, 13].

#### Contact tracing data for exposure and named contacts

All individuals testing positive for SARS-CoV-2 via PCR, including re- infections, were referred for contact tracing by NHS Test and Trace. Where individuals had multiple positive tests within 10-days, the first positive test became the case record. Symptom onset, date of birth, sex, ethnicity and address were collected during contact tracing and cases were asked to name people they had close contact with when they were infectious (from two days before symptom onset until date of contact tracing), recording setting type and exposure dates. For asymptomatic individuals, test date was used in place of symptom onset throughout.

These data were used to assess secondary attack rates in named contacts and provide proxy household size for the household clustering analysis.

#### Data linkage and processing

Vaccination status of cases and contacts was obtained by linking case data to the National Immunisation Management System (NIMS) using a unique patient identifier (NHS number) or combinations of NHS number, forename, first initial, surname, date of birth and postcode [14]. Vaccination status was derived at time of symptom onset or positive test (cases) or exposure (contacts). Vaccination status was derived from calculating time between vaccination date and date of symptom onset or positive test (cases) or exposure (contacts).

Case data were linked to NHS Test and Trace records using a combination of specimen identifiers, NHS number, and date of birth to enrich with the number of named household contacts.

Variant information was linked using specimen identifiers and dates to NHS Test and Trace data.

#### Transmission to named contacts

Named contacts of cases reported to NHS Test and Trace with exposure date between 03 and 12 December 2021 were included. Close contacts were defined as: household members, face-to-face contacts within one metre or within 2 m for 15 min [15]. Contacts not named by the case (for example, identified as part of contact tracing of international travellers on flights) and those with missing information on sex were excluded. The proportion of cases who completed contact tracing in each group was assessed.

The outcome of interest was whether an individual named as a contact became a secondary case, therefore, transmission. This was identified by matching the named contact to a PCR-positive case (secondary case) in NHS Test and Trace data with symptom onset date 2–14 days (inclusive) after exposure date. Records were matched using forename, surname and combinations of NHS number, date of birth, postcode, email or telephone number. For household contacts, symptom onset of their exposer was used as exposure date. Where multiple contact events (from multiple cases or the same case on multiple occasions) were matched to a single secondary case record, rule-based prioritisation was used to select a single contact event, prioritising household exposures and most recent exposures. The number of contacts with multiple contact events in the period was assessed.

#### Household clustering

The household clustering analyses included ‘index’ cases, the first positive test within a household. Index cases were laboratory confirmed sequenced cases identified as Omicron or Delta whereas further household cases were confirmed by any method (including LFD) regardless of sequencing status to optimise case ascertainment. Index cases were individuals with first positive specimens between 05 and 11 December 2021 and excluded re-infections. Only cases living in private dwellings (flats, terraced, semi-detached or detached houses) were included.

A household cluster was defined as two or more cases (the index case plus at least one other case) at the same private residential dwelling, with a secondary case occurring within 14 days of the first positive specimen. Sporadic cases are single cases detected in a household within a 14-day period. Residential household clusters were identified from cases' home addresses self-reported at the time of booking a COVID-19 test or from the diagnosing laboratory or NHS spine (summary care records). Residential addresses were address-matched against Ordnance Survey reference databases to derive a Unique Property Reference Number, and Basic Land and Property Unity to identify property type.

Index cases were excluded if they (i) were in households with a case with an earliest positive test in the preceding 90 days of the index case as this could reduce the number of susceptible persons in a household (ii) had co-primary household cases, defined as more than one case diagnosed within one day of each other, as household transmission could be from either case (iii) cases without named household contacts identified through contact tracing, including those who did not complete contact tracing documents. (iv) cases identified through non-community testing i.e. hospital testing, to reduce any bias by including hospitalised patients who would not contribute to household clustering.

### Descriptive analysis

For transmission among named contacts, demographic, vaccination, testing and contact tracing characteristics of contacts of Delta and Omicron cases and their exposers were described. The English Indices of Multiple Deprivation (IMD) quintiles for 2019 at Lower Super Output Area level (2011 boundaries) in England were used. Median serial intervals were calculated as the days between symptom onset of symptomatic cases (exposers) and subsequent symptomatic cases (among named contacts). The mean number of household and non-household contacts for each variant was described. Secondary attack rates, the proportion of close contacts that became cases, were calculated for Omicron and Delta.

For the household clustering analyses, Delta and Omicron index cases were described by specimen test date, age, sex, ethnicity, IMD, region, number of household contacts and vaccination status.

Where CT value data were available, the proportion of genotyped and sequenced Omicron and Delta cases with ≤30CT for both N and ORF1ab genes were assessed to evaluate any bias introduced by use of SGTF to indicate Omicron but not Delta.

### Statistical analysis

To evaluate differences in transmission to named contacts for Omicron and Delta, logistic regression models were used. Multivariable models to compare the risk of contacts becoming a case for each variant were conducted for household and non-household contacts. The models adjusted for age group, sex, vaccination status of both exposer and contact, symptom status, region of residence, IMD and ethnicity of the exposer, exposure date and whether the contact completed contact tracing. Contacts with missing information about their exposer's IMD were excluded from models. To assess differences in transmission between Omicron and Delta by vaccination status due to differential protection from vaccination, interactions between variant and vaccination status of the exposer and the contact were included in the models. Likelihood ratio tests were used to assess the model fit. To account for potential non-independence amongst contacts of the same exposer, generalised estimating equation models with the same outcome and predictor variables, the exposer as the grouping factor, and an unstructured correlation structure were fitted.

To aid interpretation, the results are presented as adjusted secondary attack rates and risk ratios. Post-estimation analyses to assess the risk ratio in transmission to named contacts for Omicron and Delta were carried out [16]. Adjusted secondary attack rates and adjusted risk ratios (aRRs) among contacts of cases where both case and contact were unvaccinated were derived from the same models.

For household clustering a logistic regression model was fitted to assess whether Omicron index cases were more likely to result in household clustering of cases compared to Delta, with the outcome as a binary indicator for clustering. The model was adjusted for age, sex, ethnicity, IMD, number of household contacts, household type (terraced, semi-detached, detached or flat), earliest positive specimen date, region, asymptomatic *vs.* symptomatic and vaccination status. Two interaction parameters were assessed to identify any effect modification between variant and specimen date and between variant and vaccination status to account for changes in PCR test availability in December 2021 and differential vaccine effectiveness by variant, respectively.

An additional model was constructed as a sensitivity analysis including only individuals without recent travel history outside the UK to consider behavioural and testing differences among recent travellers.

Statistical analyses were conducted in Stata version 15 and R version 4.0.5 [17, 18].

## Results

### Secondary attack rates

Of 23 667 Omicron and 59 031 Delta cases between 05 and 11 December 2021, 13 874 Omicron and 40 453 Delta cases named at least one contact (in any setting) during contact tracing. After excluding contacts exposed outside of 03 to 12 December 2021, and contacts without recorded sex (*n* = 54) or exposer IMD (*n* = 260) we included 40 123 Omicron and 111 469 Delta contacts in the secondary attack rate analysis, exposed by 13 680 Omicron and 37 601 Delta cases respectively. Among the cohort of 151 592 contacts, 142 527 people were identified: 95.2% (135 634) were exposed once and 4.4% (6207) exposed twice.

88% of Delta cases and 89% of Omicron cases completed contact tracing. Delta cases reported a mean of 2.0 contacts per case (s.d. (standard deviation) 2.2), 1.6 (s.d. 1.5) household and 0.4 (s.d. 1.5) non-household. Omicron cases reported fewer contacts, but with more variation; 1.7 contacts per case (s.d. 2.9), 1.1 household (s.d. 1.3) and 0.6 (s.d. 2.4) non-household.

Within households, the median serial interval from exposer to secondary case was 4 days (IQR (interquartile range): 2–6 days) for Delta and 3 days (IQR: 2–5) for Omicron. This was similar outside the household, with median serial interval 4 days (IQR: 2–7) for Delta and 3 days (IQR: 1–5) for Omicron.

The unadjusted secondary attack rate among named Omicron household contacts was 15.0% (3812) compared to 10.8% (9564) for Delta contacts. Similarly, secondary attack rates among non-household contacts were higher for Omicron than Delta, 8.2% (1212) *vs.* 3.7% (850).

Logistic regression models were fitted. Data in the household model were consistent with interactions of variant with exposer vaccination status and variant with contact vaccination status (*P* ≤ 0.00033); as these were plausible, they were maintained in both models. Coefficients from generalised estimating equation models were very close to those from the logistic regression model, with only slightly larger standard errors (at most 0.084 greater than those estimated in the logistic regression model); as these didn't change our inference, the results from the standard logistic regression model are presented.

The overall aRR of transmission to household contacts for Omicron compared to Delta cases was 1.48 (95% confidence interval (CI) 1.41–1.55) and 2.14 (95% CI 1.91–2.40) for non-household contacts.

Although the effect of variant on transmission varied by vaccination status (Table 1), adjusted secondary attack rates were consistently higher for Omicron for every stratum of vaccination dose for exposers and contacts.

**Table 1.** Adjusted^a^ secondary attack rates and adjusted risk ratios of transmission to named contacts from Omicron compared to Delta cases in household (1A) and non-household (1B) settings

	1A: Household			*P*
	Adjusted secondary attack rate Delta (95% CI)	Adjusted secondary attack rate Omicron (95% CI)	Adjusted risk ratio of Omicron vs. Delta (95% CI)	
All	10.5% (10.3%–10.7%)	15.6% (15.0%–16.1%)	1.48 (1.41–1.55)	<0.0001
Contact vaccination status				
Unvaccinated	12.9% (12.4%–13.5%)	15.9% (14.8%–17.0%)	1.23 (1.14–1.32)	<0.0001
1 dose + 21 days	10.6% (9.7%–11.5%)	14.8% (12.8%–16.8%)	1.40 (1.19–1.63)	<0.0001
2 doses + 14 days	11.2% (10.8%–11.6%)	18.3% (17.4%–19.3%)	1.64 (1.55–1.75)	<0.0001
3 doses + 14 days	7.6% (6.9%–8.3%)	16.1% (14.5%–17.7%)	2.13 (1.87–2.43)	<0.0001
Exposer vaccination status				
Unvaccinated	11.8% (11.4%–12.3%)	15.8% (14.7%–17.0%)	1.33 (1.23–1.44)	<0.0001
1 dose + 21 days	9.6% (8.8%–10.4%)	14.9% (13.1%–16.8%)	1.55 (1.34–1.81)	<0.0001
2 doses + 14 days	10.0% (9.6%–10.3%)	15.8% (15.1%–16.5%)	1.58 (1.50–1.67)	<0.0001
3 doses + 14 days	6.2% (5.3%–7.1%)	12.4% (10.9%–13.8%)	1.99 (1.66–2.39)	<0.0001

a with additional adjustment for exposure date, characteristics of index cases (age, sex, IMD and ethnicity, symptom status, region of residence) and contacts (age group, sex, whether they completed contact tracing).

**Table:** 

	1B: Non-household			*P*
	Adjusted secondary attack rate Delta (95% CI)	Adjusted secondary attack rate Omicron (95% CI)	Adjusted risk ratio of Omicron vs. Delta (95% CI)	
All	3.9% (3.7%–4.2%)	7.6% (7.2%–8.1%)	2.14 (1.91–2.40)	<0.0001
Contact vaccination status				
Unvaccinated	5.1% (3.4%–6.8%)	10.1% (7.4%–12.8%)	1.99 (1.32–2.99)	0.0009
1 dose + 21 days	6.0% (3.2%–8.9%)	7.4% (3.8%–11.0%)	1.22 (0.62–2.39)	0.56
2 doses + 14 days	5.9% (5.1%–6.7%)	9.6% (8.4%–10.9%)	1.64 (1.43–1.87)	<0.0001
3 doses + 14 days	3.0% (2.2%–3.8%)	7.3% (5.7%–9.0%)	2.43 (1.80–3.29)	<0.0001
Exposer vaccination status				
Unvaccinated	4.9% (4.0%–5.8%)	8.8% (7.2%–10.4%)	1.33 (1.23–1.44)	<0.0001
1 dose + 21 days	4.7% (3.2%–6.3%)	6.7% (4.6%–8.9%)	1.55 (1.34–1.81)	<0.0001
2 doses + 14 days	3.7% (3.4%–4.1%)	7.5% (6.9%–8.0%)	1.58 (1.50–1.67)	<0.0001
3 doses + 14 days	3.1% (2.1%–4.2%)	7.1% (5.7%–8.4%)	1.99 (1.66–2.39)	<0.0001

a with additional adjustment for exposure date, characteristics of index cases (age, sex, IMD and ethnicity, symptom status, region of residence) and contacts (age group, sex, whether the completed contact tracing).

Exposer and contacts with unknown vaccination status omitted.

For Delta cases, adjusted secondary attack rates were lowest among exposers and contacts with three doses of vaccine in both settings (Tables 1a and 1b). For Delta contacts, secondary attack rates were 3.0% for individuals with three doses *vs.* 5.1% in unvaccinated contacts in non-household settings, and 7.6% *vs.* 12.9% in household settings. Similarly, transmission was reduced when exposers had three doses, 3.1% *vs.* 4.9% for no doses in non-household settings and 6.2% *vs.* 11.8% in households. The aRR of transmission for contacts who had received a third vaccine compared to two doses was 0.51 for non-household and 0.68 for household contacts (Supplementary Table 1B).

The reduction of transmission associated with exposer or contact vaccination for Omicron was considerably attenuated compared to Delta. Household secondary transmission rates were more similar across vaccination strata, although lower where exposers had 3 doses (12.4%) compared to less than 3 doses or unvaccinated (14.9 to 15.8%; Table 1). In non-household settings, a protective effect for contacts having received 3 doses *vs.* 2 doses was observed (aRR = 0.76), but there was no evidence of differences in protection according to number of doses received by exposers (Supplementary Table 1B).

Where both exposer and contact were unvaccinated, adjusted secondary attack rates in households were slightly higher for Omicron (16.2%, 95% CI 14.8%–17.6%, *n* = 1835) compared to Delta (14.6%, 95% CI 13.9%–15.3%, *n* = 15 035) with aRR of 1.11 (95% CI 1.01–1.22). For non-household contacts the difference between secondary attack rates was more marked, with 11.6% (95% CI 8.2%–14.9%, *n* = 124) for Omicron and 6.3% (95% CI 4.1%–8.6%, *n* = 209) for Delta and aRR of 1.84 (95% CI 1.19–2.85).

**Fig. 1.**
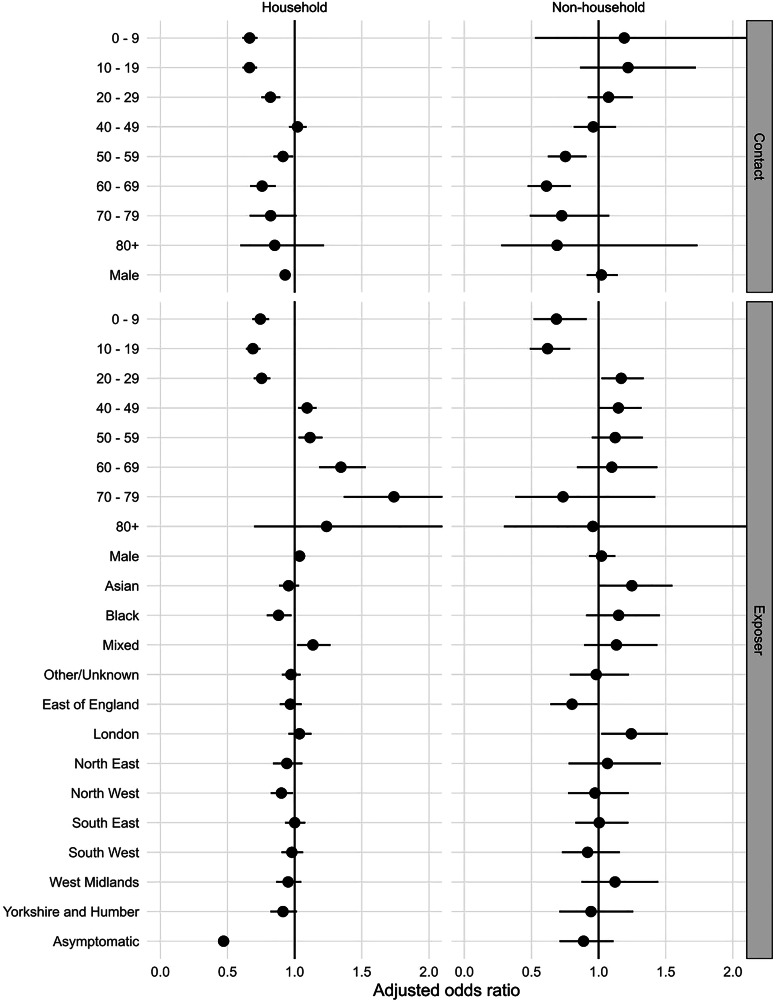
Transmission to named contacts: adjusted odds ratios for selected variables* from multivariable analyses (*x*-axis limited to 2), 05 to 11 December 2021, England. *with additional adjustment for variant, exposer vaccination status, contact vaccination status, interaction of variant with exposer vaccination status, interaction of variant with contact vaccination status, whether contact completed contact tracing, exposer IMD quintile, date of exposure. Missing values omitted for all categories.

Compared to 30–39-year-olds, transmission risk was lower for contacts of all other ages except 40–49 years, particularly children (Fig. 1). Likelihood of transmission was also lower for male contacts. Household exposers aged under 30 were less likely to transmit than those aged 30–79 years old. Similarly, outside the household, exposers aged under 20 were less likely to transmit than 30–69-year-olds. Transmission was more likely to non-household contacts of exposers in London than in the reference region (East Midlands), and less likely to household contacts in the North West. Within households, asymptomatic cases (13.5% (11 986) of Delta, 8.8% (2247) of Omicron) were half as likely to transmit to their household contacts (aOR 0.47) than those reporting symptoms, but no evidence of difference was seen for non-household exposures.

Quantifying any bias introduced by selection of Omicron cases via SGTF, of those identified by sequencing or genotyping with CT value data, 98.7% (20 400) of Delta cases and 99.2% (3259) of Omicron cases had CT≤30 for both N and ORF1ab genes.

#### Household clustering

From 05 to 11 December 2021, a total of 307 034 individuals tested positive for SARS-CoV-2 for the first time of which 60 393 cases were confirmed as Delta by genotyping or sequencing and 21 402 identified as Omicron through genomic sequencing or the presence of SGTF, representing a total of 26.6% of all cases reported in England.

After exclusion criteria were applied, 8692 Omicron and 29 094 Delta index cases were included in the household clustering analysis.

Of the cases included in the analysis, 16.1% (1404) Omicron cases resulted in household clustering, compared to 7.3% (2136) Delta cases.

The multivariable logistic regression model showed a significant effect modification between variant and the vaccination status of the index case and this interaction was retained in the final model.

Post-estimation analysis to assess the risk ratio of household clustering found overall risk ratio of 3.54 for Omicron compared to Delta variant. Furthermore, for each vaccination status there was an increased risk of household clustering for Omicron compared to Delta variant, most notably among index cases who had had a third vaccine dose, with a risk ratio of household clustering of 6.81 (Table 2).

**Table 2.** Risk of household clustering for Omicron and Delta by vaccination status of the index case

Vaccination status of index case	Adjusted risk of clustering, Delta (%)	Adjusted risk of clustering, Omicron (%)	Adjusted risk ratio (aRR) Omicron vs. Delta
All	6.58 (6.31–6.86)	23.32 (21.97–24.67)	3.54 (3.29–3.81)
Unvaccinated	7.79 (7.14–8.45)	24.83 (22.04–27.62)	3.19 (2.79–3.64)
≥21 days post dose 1	6.10 (4.98–7.22)	24.93 (20.55–29.31)	4.09 (3.19–5.23)
≥14 days post dose 2	6.19 (5.73–6.64)	22.02 (20.55–23.5)	3.56 (3.25–3.90)
≥14 days post dose 3	3.05 (2.13–3.98)	20.80 (17.64–23.97)	6.81 (4.91–9.46)
Unknown vaccination status	6.80 (5.35–8.25)	27.54 (22.49–32.59)	4.05 (3.06–5.37)

Effect modification between specimen date and variant was evaluated and identified as not being significant (*P* = 0.123).

**Fig. 2.**
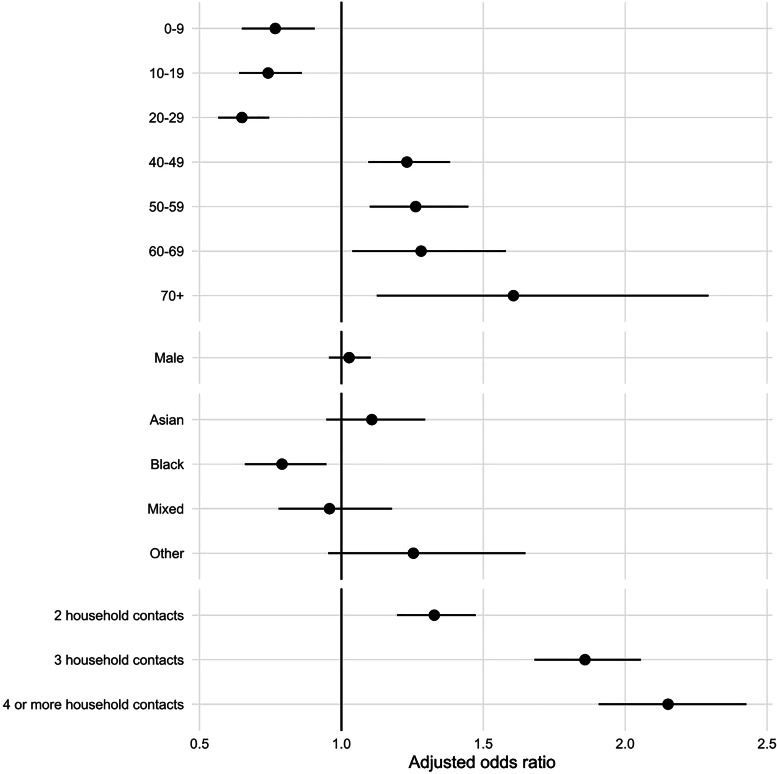
Household clustering for selected variables from multivariable analyses: adjusted odds ratios, 5 to 11 December 2021, England*. *The full adjusted model includes adjustment for variant (Omicron and Delta), sex, age group, ethnicity, IMD, household type, earliest specimen date, region, vaccination status, number of household contacts, symptomatic status.

Additional factors associated with likelihood of household clustering were age, with younger (<30 years old) index cases having the lowest likelihood of household clustering compared to 30–39-year-olds, and those over 40 years having higher risk, and ethnicity, with reduced household clustering for black *vs.* white index cases (Fig. 2).

The overall results of the model for household clustering did not significantly change in a restricted model excluding those with travel history outside of the UK showing an overall aRR of 3.55 (95% CI 3.30–3.83)), compared to 3.54 (95% CI 3.29–3.81) in the final model including those with travel history.

## Discussion

We found increased transmission risk for Omicron compared to Delta in households by using complementary but distinct methods to assess transmission to named contacts through contact tracing and household clustering surveillance data. These two analytical methods use separate data sources with different uses and data collection methods. This allowed for transmissibility within both household and non-household setting to be assessed and added rigour to our assessment to answer an important public health question.

We identified a greater transmission risk from Omicron cases to their close contacts in non-household settings, with Omicron contacts twice as likely to develop infection than Delta.

We identified a significant attenuation of the protective effect of a third vaccination dose in reducing risk on onwards transmission for Omicron cases. This was likely to have contributed the acceleration in incidence following the arrival of Omicron in November 2021, exceeding 2000 cases per 100 000 population by 04 January 2022. This triggered public health measures to be reinstated including compulsory face coverings, proof of vaccination in most public indoor venues and encouragement to work from home [19].

The domination of Omicron globally highlights the importance of rapid genomic surveillance to detect and better understand the impact of new variants on disease incidence, hospitalisations and mortality. With evidence of waning immunity, public campaigns strongly encouraged a third dose (booster) of vaccine for all adults. We found a modest benefit in reducing transmission of Omicron and greater impact for Delta. However, substantial benefits to patient outcomes, including reduced severity of disease, were achieved, alleviating the burden of rising case numbers on the health service through the booster immunisation programme [20, 21].

The observed increased transmission risk for Omicron compared to Delta among unvaccinated contacts of unvaccinated Omicron cases suggests that Omicron has intrinsic properties that aid transmission [22]. The observed increased transmission of Omicron (relative to Delta) in non-household settings (compared to household settings) across all vaccination groups suggest that less proximate contact may be sufficient for transmission from Omicron cases.

After accounting for variant and vaccination status, the risk of transmission to named contacts of both Omicron and Delta cases was reduced for those with a third vaccination dose, supporting vaccine effectiveness findings which indicate increased effectiveness of a third dose of vaccine against Omicron among individuals with a third vaccination dose [20]. Vaccination records on the NIMS have previously shown over 99% of records agree with self-reported vaccination status acquired through an enhanced surveillance questionnaire, therefore we do not anticipate vaccination records impacting the results observed [14]. However, the relative impact was less compared to Delta. An index case having a third dose did not substantially decrease the risk of household clustering where the index case had Omicron, whereas a third dose significantly reduced household clustering arising from Delta. These findings align with initial findings from Denmark, Norway and the Netherlands [23–26].

This is one of the first studies to investigate Omicron transmission in both household and non-household settings. Having a robust national-level dataset and being able to link individual cases by address to secondary cases, as well as through named household contacts identified via contact tracing, has allowed for two complementary methods to assess household transmission in England. Routine collection of named contacts outside the household in national contact tracing of all cases allowed the evaluation of transmission in these settings. Through linkage of national immunisation datasets to both cases and contacts, we were able to robustly assess the reduction in transmission from vaccination and evaluate the effect of different doses, on risk, both for onward transmission from the index and for susceptibility of the contact.

The fourth wave of COVID-19 resulted in considerable demands for tests and shortages in supply and as such, it is likely not all clusters or transmission events were detected [27]. Furthermore, it is possible that behaviours changed closer to the Christmas period which may have suppressed transmission rates observed. However, this is unlikely to have differentially affected Omicron compared to Delta cases. Transmission estimates from routinely collected data should be considered lower bounds due to limitations of data completeness and quality from variation in testing behaviour and engagement with contact tracing. Positive tests on LFDs without confirmatory PCR were not included in the secondary attack rate analysis. Our assessment of the effect of vaccination on transmission did not consider the timing of the vaccinations received and so could not distinguish the effect of multiple doses from recency of the vaccination. Children under 12 were not eligible for COVID-19 vaccination at the time of the study. Although models accounted for age and vaccination status of exposer and contact separately, the full effect of unvaccinated children on transmission rates within their households may not be fully accounted for.

In summary, we identified increased risk of transmission from Omicron compared to Delta, in part explained by an attenuated impact of vaccination on reducing transmission for Omicron compared to Delta. As such, Omicron's worldwide success is likely to be at least in part attributable to immune escape. Our findings underscore the value of assessing growth advantage of new variants as they emerge to inform roll-out of booster vaccinations and other non-pharmaceutical public health interventions.

## Supplementary material

For supplementary material accompanying this paper visit https://doi.org/10.1017/S0950268823000420. click here to view supplementary material

## Data sharing statement

The data that support these studies were collected as part of a public health response, are considered sensitive and not made publicly available. Reasonable requests for access to anonymised data and data dictionary will be considered by the authors on request to allow all results to be reproduced.
