# Supplementary material for: Comparative transmission of SARS-CoV-2 Omicron (B.1.1.529) and Delta (B.1.617.2) variants and the impact of vaccination: national cohort study, England
Source: Epidemiol Infect. 2023 Mar 20;151:e58. doi: 10.1017/S0950268823000420 (PMC10125873; doi:10.1017/S0950268823000420)
Supplement: Supplementary file 1 [file S0950268823000420sup001.docx]

**SUPPLEMENTARY TABLES**

Table S1A. Adjusted risk ratios of vaccination on transmission to household contacts of cases with Omicron and Delta. Reference category is vaccinated with 2 doses + 14 days.

| Household | | | | |
| --- | --- | --- | --- | --- |
| Vaccination status | Adjusted risk ratio of vaccination status on Delta cases | p | Adjusted risk ratio of vaccination status on Omicron cases | p |
| Exposer | | | | |
| Unvaccinated | 1.19 (1.12 - 1.27) | <0.0001 | 1.00 (0.92 - 1.09) | 0.9323 |
| 1 dose + 21 days | 0.96 (0.87 - 1.06) | 0.4550 | 0.94 (0.83 - 1.08) | 0.4062 |
| 3 doses + 14 days | 0.62 (0.54 - 0.72) | <0.0001 | 0.78 (0.69 - 0.88) | <0.0001 |
| Contact | | | | |
| Unvaccinated | 1.16 (1.08 - 1.24) | <0.0001 | 0.87 (0.79 - 0.95) | 0.0015 |
| 1 dose + 21 days | 0.95 (0.86 - 1.04) | 0.2627 | 0.80 (0.69 - 0.93) | 0.0029 |
| 3 doses + 14 days | 0.68 (0.62 - 0.74) | <0.0001 | 0.88 (0.79 - 0.97) | 0.0129 |

Adjusted as in main model for exposure date, characteristics of index cases and contacts (age group, sex), whether the contact completed contact tracing and the symptom status, region of residence, IMD and ethnicity of the index case.

Table S1B: Adjusted risk ratios of vaccination on transmission to non-household contacts of cases with Omicron and Delta. Reference category is vaccinated with 2 doses + 14 days.

| Non-household | | | | |
| --- | --- | --- | --- | --- |
| Vaccination status | Adjusted risk ratio of vaccination status on Delta cases | p | Adjusted risk ratio of vaccination status on Omicron cases | p |
| Exposer | | | | |
| Unvaccinated | 1.32 (1.06 - 1.65) | 0.0125 | 1.18 (0.97 - 1.44) | 0.1044 |
| 1 dose + 21 days | 1.28 (0.9 - 1.81) | 0.1676 | 0.9 (0.64 - 1.26) | 0.5434 |
| 3 doses + 14 days | 0.84 (0.59 - 1.19) | 0.3320 | 0.95 (0.77 - 1.16) | 0.6042 |
| Contact | | | | |
| Unvaccinated | 0.86 (0.61 - 1.21) | 0.3960 | 1.05 (0.8 - 1.36) | 0.7299 |
| 1 dose + 21 days | 1.02 (0.63 - 1.66) | 0.9218 | 0.76 (0.47 - 1.25) | 0.2829 |
| 3 doses + 14 days | 0.51 (0.39 - 0.66) | <0.0001 | 0.76 (0.61 - 0.94) | 0.0115 |

Adjusted as in main model for exposure date, characteristics of index cases and contacts (age group, sex), whether the contact completed contact tracing and the symptom status, region of residence, IMD and ethnicity of the index case.

Table S2. Descriptive analysis of household clustering, and results of multiple regression model of transmission in household clusters

| **Variable** | | **Omicron/ Total cases (% Omicron)** | **Univariable OR (95% CI)** | **Multivariable OR (95% CI)** |
| --- | --- | --- | --- | --- |
| **Variant** | **Delta** | - | Baseline | |
|  | **Omicron** | 8692 / 37786 (23.00) | 2.43 (2.26 to 2.61) | * |
| **Sex** | **Female** | 4702 / 20065 (23.43) | Baseline | |
|  | **Male** | 3990 / 17717 (22.52) | 1.05 (0.98 to 1.12) | 1.03 (0.96 to 1.10) |
| **Age Group** | **30-39** | 1977 / 6425 (30.77) | Baseline | |
|  | **<10** | 259 / 5653 (4.58) | 0.83 (0.74 to 0.95) | 0.77 (0.65 to 0.91) |
|  | **10-19** | 1085 / 7546 (14.38) | 0.88 (0.78 to 0.98) | 0.74 (0.64 to 0.86) |
|  | **20-29** | 2486 / 4767 (52.15) | 0.80 (0.70 to 0.91) | 0.65 (0.57 to 0.75) |
|  | **40-49** | 1507 / 6992 (21.55) | 1.17 (1.05 to 1.30) | 1.23 (1.10 to 1.38) |
|  | **50-59** | 954 / 4333 (22.02) | 1.02 (0.89 to 1.16) | 1.26 (1.10 to 1.45) |
|  | **60-69** | 326 / 1530 (21.31) | 0.87 (0.72 to 1.06) | 1.28 (1.04 to 1.58) |
|  | **70+** | 98 / 536 (18.28) | 0.78 (0.56 to 1.08) | 1.61 (1.13 to 2.29) |
| **Ethnicity** | **White** | 6616 / 32457 (20.38) | Baseline | |
|  | **Mixed** | 348 / 1213 (28.69) | 0.98 (0.81 to 1.20) | 0.96 (0.78 to 1.18) |
|  | **Asian** | 537 / 2011 (26.7) | 1.11 (0.96 to 1.29) | 1.11 (0.95 to 1.29) |
|  | **Black** | 1009 / 1537 (65.65) | 1.25 (1.06 to 1.47) | 0.79 (0.66 to 0.95) |
|  | **Other** | 182 / 564 (32.27) | 1.26 (0.97 to 1.64) | 1.25 (0.95 to 1.65) |
| **IMD Quintile** | **3** | 1686 / 7552 (22.33) | Baseline | |
|  | **1 - most deprived** | 1128 / 5019 (22.47) | 0.98 (0.87 to 1.11) | 1.04 (0.92 to 1.19) |
|  | **2** | 1811 / 6945 (26.08) | 1.07 (0.96 to 1.20) | 1.08 (0.96 to 1.21) |
|  | **4** | 1867 / 8252 (22.62) | 0.96 (0.86 to 1.07) | 0.97 (0.86 to 1.08) |
|  | **5- least deprived** | 2200 / 10014 (21.97) | 1.08 (0.98 to 1.20) | 1.08 (0.97 to 1.21) |
| **Household type** | **Detached** | 1931 / 9861 (19.58) | Baseline | |
|  | **Terraced** | 2562 / 11417 (22.44) | 1.12 (1.02 to 1.23) | 1.2 (1.09 to 1.33) |
|  | **Semi-Detached** | 2321 / 11946 (19.43) | 1.05 (0.96 to 1.15) | 1.15 (1.04 to 1.27) |
|  | **Flat** | 1878 / 4558 (41.2) | 0.99 (0.87 to 1.12) | 1.07 (0.93 to 1.24) |
| **Earliest Specimen Date** | **05/12/2021** | 173 / 4609 (3.75) | Baseline | |
|  | **06/12/2021** | 456 / 5823 (7.83) | 0.93 (0.81 to 1.05) | 0.91 (0.80 to 1.03) |
|  | **07/12/2021** | 773 / 5708 (13.54) | 1.05 (0.93 to 1.19) | 0.92 (0.81 to 1.05) |
|  | **08/12/2021** | 1207 / 5410 (22.31) | 1.03 (0.91 to 1.18) | 0.80 (0.70 to 0.92) |
|  | **09/12/2021** | 1568 / 5429 (28.88) | 0.91 (0.80 to 1.03) | 0.63 (0.54 to 0.72) |
|  | **10/12/2021** | 2270 / 5766 (39.37) | 0.77 (0.67 to 0.88) | 0.45 (0.39 to 0.52) |
|  | **11/12/2021** | 2245 / 5037 (44.57) | 0.57 (0.49 to 0.66) | 0.30 (0.26 to 0.35) |
| **Region** | **London** | 2903 / 5886 (49.32) | Baseline | |
|  | **East Midlands** | 562 / 3099 (18.13) | 0.81 (0.70 to 0.95) | 1.14 (0.96 to 1.35) |
|  | **East of England** | 1323 / 4767 (27.75) | 0.96 (0.84 to 1.09) | 1.13 (0.98 to 1.30) |
|  | **North East** | 119 / 1579 (7.54) | 0.77 (0.63 to 0.94) | 1.21 (0.97 to 1.51) |
|  | **North West** | 1016 / 3508 (28.96) | 0.85 (0.74 to 0.99) | 1.00 (0.85 to 1.17) |
|  | **South East** | 1694 / 9201 (18.41) | 0.97 (0.87 to 1.09) | 1.28 (1.13 to 1.45) |
|  | **South West** | 351 / 5283 (6.64) | 1.15 (1.02 to 1.29) | 1.92 (1.67 to 2.21) |
|  | **West Midlands** | 449 / 2507 (17.91) | 0.72 (0.61 to 0.86) | 1.05 (0.87 to 1.26) |
|  | **Yorkshire and Humber** | 275 / 1952 (14.09) | 0.69 (0.57 to 0.84) | 1.02 (0.83 to 1.25) |
| **Vaccination status** | **>=14 Days post dose 2** | 5628 / 18704 (30.09) | Baseline | |
|  | **>=14 Days post dose 3/ booster** | 843 / 2093 (40.28) | 0.85 (0.73 to 1.00) | * |
|  | **>=21 Days post dose 1** | 480 / 2329 (20.61) | 0.85 (0.73 to 0.99) |  |
|  | **Unknown/ unlinked** | 372 / 1463 (25.43) | 1.04 (0.87 to 1.24) |  |
|  | **Unvaccinated** | 1369 / 13193 (10.38) | 0.88 (0.81 to 0.95) |  |
| **Number of household contacts** | **1 household contact** | 4006 / 12789 (31.32) | Baseline | |
|  | **2 household contacts** | 2092 / 8908 (23.48) | 1.17 (1.06 to 1.29) | 1.33 (1.20 to 1.47) |
|  | **3 household contacts** | 1858 / 11031 (16.84) | 1.50 (1.37 to 1.64) | 1.86 (1.68 to 2.06) |
|  | **4 or more household contacts** | 736 / 5054 (14.56) | 1.65 (1.48 to 1.83) | 2.15 (1.91 to 2.43) |
| **Symptomatic status** | **Symptomatic** | 15734 / 20516 (76.69) | Baseline | |
|  | **Unknown** | 1454 / 1605 (90.59) | 0.77 (0.63 to 0.93) | 0.92 (0.76 to 1.13) |
|  | **Asymptomatic** | 11906 / 15665 (76.00) | 1.02 (0.95 to 1.10) | 1.02 (0.95 to 1.10) |

*Multivariable not reported due to effect modification with omicron

Table S3. Descriptive analysis of household contacts and their exposing cases, and results of household model of transmission to named contacts

| Household | | | | | |
| --- | --- | --- | --- | --- | --- |
|  |  | Delta | Omicron | OR (univariable) | OR (multivariable) |
| Variant | Delta | 88831 (77.8) |  | - | - |
|  | Omicron |  | 25401 (22.2) | 1.46 (1.41-1.52, p<0.001) | * |
| Exposer Age | 0 - 9 | 21009 (23.7) | 1320 (5.2) | 0.72 (0.68-0.77, p<0.001) | 0.74 (0.68-0.81, p<0.001) |
|  | 10 - 19 | 23135 (26.0) | 4492 (17.7) | 0.69 (0.65-0.73, p<0.001) | 0.69 (0.64-0.74, p<0.001) |
|  | 20 - 29 | 5080 (5.7) | 6351 (25.0) | 0.81 (0.75-0.87, p<0.001) | 0.75 (0.70-0.82, p<0.001) |
|  | 30 - 39 | 12831 (14.4) | 5018 (19.8) | - | - |
|  | 40 - 49 | 17111 (19.3) | 4785 (18.8) | 1.03 (0.97-1.09, p=0.301) | 1.09 (1.03-1.16, p=0.005) |
|  | 50 - 59 | 7137 (8.0) | 2612 (10.3) | 1.04 (0.96-1.11, p=0.337) | 1.11 (1.03-1.21, p=0.007) |
|  | 60 - 69 | 1916 (2.2) | 635 (2.5) | 1.12 (1.00-1.26, p=0.051) | 1.34 (1.18-1.53, p<0.001) |
|  | 70 - 79 | 483 (0.5) | 163 (0.6) | 1.20 (0.97-1.49, p=0.097) | 1.74 (1.37-2.21, p<0.001) |
|  | 80+ | 129 (0.1) | 25 (0.1) | 0.70 (0.41-1.19, p=0.190) | 1.24 (0.70-2.19, p=0.465) |
| Exposer Sex | Female | 46844 (52.7) | 13992 (55.1) | - | - |
|  | Male | 41987 (47.3) | 11409 (44.9) | 1.04 (1.00-1.08, p=0.040) | 1.04 (1.00-1.08, p=0.060) |
| Exposer Ethnicity | Asian | 5615 (6.3) | 1904 (7.5) | 0.92 (0.85-0.99, p=0.027) | 0.96 (0.88-1.03, p=0.248) |
|  | Black | 1447 (1.6) | 2908 (11.4) | 0.94 (0.85-1.03, p=0.187) | 0.88 (0.79-0.98, p=0.016) |
|  | Mixed | 2236 (2.5) | 945 (3.7) | 1.10 (0.99-1.22, p=0.080) | 1.14 (1.02-1.27, p=0.021) |
|  | Other/Unknown | 7194 (8.1) | 2117 (8.3) | 0.90 (0.84-0.96, p=0.002) | 0.97 (0.91-1.04, p=0.442) |
|  | White | 72339 (81.4) | 17527 (69.0) | - | - |
| Exposer Region | East Midlands | 7206 (8.1) | 1683 (6.6) | - | - |
|  | East Of England | 10273 (11.6) | 3959 (15.6) | 1.01 (0.93-1.09, p=0.905) | 0.97 (0.89-1.05, p=0.443) |
|  | London | 8922 (10.0) | 8201 (32.3) | 1.11 (1.03-1.20, p=0.010) | 1.04 (0.95-1.12, p=0.395) |
|  | Missing | 506 (0.6) | 229 (0.9) | 1.05 (0.84-1.32, p=0.674) | 1.17 (0.91-1.52, p=0.226) |
|  | North East | 4202 (4.7) | 356 (1.4) | 0.89 (0.79-0.99, p=0.036) | 0.94 (0.84-1.06, p=0.311) |
|  | North West | 6936 (7.8) | 2937 (11.6) | 0.95 (0.87-1.04, p=0.228) | 0.90 (0.82-0.99, p=0.026) |
|  | South East | 24434 (27.5) | 4863 (19.1) | 1.00 (0.93-1.08, p=0.950) | 1.00 (0.93-1.08, p=0.977) |
|  | South West | 15551 (17.5) | 921 (3.6) | 0.93 (0.85-1.00, p=0.065) | 0.98 (0.90-1.06, p=0.622) |
|  | West Midlands | 6138 (6.9) | 1371 (5.4) | 0.93 (0.84-1.02, p=0.120) | 0.95 (0.86-1.05, p=0.320) |
|  | Yorkshire And Humber | 4663 (5.2) | 881 (3.5) | 0.89 (0.80-0.99, p=0.030) | 0.91 (0.82-1.02, p=0.101) |
| Exposer IMD | 1 - most deprived | 12405 (14.0) | 3453 (13.6) | 0.90 (0.85-0.96, p=0.002) | 0.96 (0.90-1.03, p=0.224) |
|  | 2 | 15562 (17.6) | 5254 (20.7) | 0.99 (0.93-1.05, p=0.651) | 0.99 (0.94-1.05, p=0.827) |
|  | 3 | 18048 (20.4) | 4715 (18.6) | - | - |
|  | 4 | 19106 (21.5) | 5362 (21.2) | 1.01 (0.96-1.07, p=0.702) | 1.00 (0.94-1.05, p=0.898) |
|  | 5 - least deprived | 23546 (26.6) | 6557 (25.9) | 0.99 (0.94-1.04, p=0.727) | 0.97 (0.92-1.02, p=0.245) |
| Exposer Vaccination | Unlinked | 3495 (3.9) | 1273 (5.0) | 0.76 (0.69-0.84, p<0.001) | * |
|  | Unvaccinated | 42484 (47.8) | 5287 (20.8) | 0.77 (0.74-0.80, p<0.001) | * |
|  | 1 Dose + 21 Days | 5994 (6.7) | 1706 (6.7) | 0.66 (0.61-0.72, p<0.001) | * |
|  | 2 Doses + 14 Days | 34266 (38.6) | 15144 (59.6) | - | * |
|  | 3 Doses + 14 Days | 2592 (2.9) | 1991 (7.8) | 0.75 (0.68-0.82, p<0.001) | * |
| Exposer Had Symptoms | True | 76845 (86.5) | 23154 (91.2) | - | - |
|  | False | 11986 (13.5) | 2247 (8.8) | 0.40 (0.37-0.43, p<0.001) | 0.47 (0.44-0.51, p<0.001) |
| Date of Exposure | 2021-12-03 | 6735 (7.6) | 520 (2.0) | 1.36 (1.25-1.48, p<0.001) | 1.41 (1.29-1.53, p<0.001) |
|  | 2021-12-04 | 10112 (11.4) | 984 (3.9) | 1.22 (1.13-1.31, p<0.001) | 1.23 (1.14-1.33, p<0.001) |
|  | 2021-12-05 | 14409 (16.2) | 2001 (7.9) | 1.07 (0.99-1.14, p=0.069) | 1.13 (1.05-1.21, p=0.001) |
|  | 2021-12-06 | 15892 (17.9) | 3510 (13.8) | 1.03 (0.96-1.10, p=0.465) | 1.06 (0.99-1.13, p=0.110) |
|  | 2021-12-07 | 13216 (14.9) | 3971 (15.6) | 0.94 (0.88-1.01, p=0.078) | 0.96 (0.89-1.03, p=0.216) |
|  | 2021-12-08 | 10808 (12.2) | 3971 (15.6) | - | - |
|  | 2021-12-09 | 8418 (9.5) | 4390 (17.3) | 0.92 (0.85-0.99, p=0.029) | 0.91 (0.84-0.98, p=0.013) |
|  | 2021-12-10 | 5974 (6.7) | 4004 (15.8) | 0.88 (0.81-0.96, p=0.002) | 0.88 (0.81-0.96, p=0.003) |
|  | 2021-12-11 | 3043 (3.4) | 1904 (7.5) | 0.78 (0.70-0.87, p<0.001) | 0.88 (0.79-0.98, p=0.023) |
|  | 2021-12-12 | 224 (0.3) | 146 (0.6) | 0.53 (0.35-0.80, p=0.003) | 0.46 (0.30-0.70, p<0.001) |
| Contact Completed | True | 82731 (93.1) | 22034 (86.7) | - | - |
|  | False | 6100 (6.9) | 3367 (13.3) | 0.56 (0.52-0.61, p<0.001) | 0.95 (0.76-1.19, p=0.656) |
| Contact Age | 0 - 9 | 19112 (21.5) | 3568 (14.0) | 0.76 (0.71-0.81, p<0.001) | 0.66 (0.61-0.72, p<0.001) |
|  | 10 - 19 | 18030 (20.3) | 4141 (16.3) | 0.73 (0.69-0.78, p<0.001) | 0.66 (0.61-0.72, p<0.001) |
|  | 20 - 29 | 4955 (5.6) | 3457 (13.6) | 0.92 (0.85-1.00, p=0.046) | 0.82 (0.75-0.89, p<0.001) |
|  | 30 - 39 | 13007 (14.6) | 3179 (12.5) | - | - |
|  | 40 - 49 | 17468 (19.7) | 3430 (13.5) | 0.98 (0.92-1.04, p=0.427) | 1.02 (0.96-1.09, p=0.508) |
|  | 50 - 59 | 7215 (8.1) | 3134 (12.3) | 0.95 (0.88-1.02, p=0.167) | 0.91 (0.84-0.99, p=0.026) |
|  | 60 - 69 | 2246 (2.5) | 1086 (4.3) | 0.84 (0.74-0.94, p=0.002) | 0.76 (0.67-0.86, p<0.001) |
|  | 70 - 79 | 813 (0.9) | 277 (1.1) | 0.83 (0.68-1.00, p=0.049) | 0.82 (0.67-1.01, p=0.067) |
|  | 80+ | 250 (0.3) | 89 (0.4) | 0.77 (0.54-1.08, p=0.132) | 0.85 (0.60-1.22, p=0.380) |
|  | Missing | 5735 (6.5) | 3040 (12.0) | 0.45 (0.41-0.49, p<0.001) | 0.83 (0.63-1.10, p=0.196) |
| Contact Sex | Female | 41304 (46.5) | 11686 (46.0) | - | - |
|  | Male | 39666 (44.7) | 10205 (40.2) | 0.92 (0.89-0.96, p<0.001) | 0.93 (0.89-0.97, p<0.001) |
|  | Missing | 7714 (8.7) | 3458 (13.6) | 0.55 (0.51-0.60, p<0.001) | 0.89 (0.78-1.02, p=0.103) |
|  | Not Specified | 147 (0.2) | 52 (0.2) | 0.65 (0.39-1.06, p=0.086) | 0.75 (0.45-1.24, p=0.267) |
| Contact Vaccination | Unlinked | 16938 (19.1) | 6500 (25.6) | 0.45 (0.42-0.47, p<0.001) | * |
|  | Unvaccinated | 30119 (33.9) | 5827 (22.9) | 0.82 (0.79-0.86, p<0.001) | * |
|  | 1 Dose + 21 Days | 5581 (6.3) | 1437 (5.7) | 0.69 (0.63-0.75, p<0.001) | * |
|  | 2 Doses + 14 Days | 30413 (34.2) | 9177 (36.1) | - | * |
|  | 3 Doses + 14 Days | 5780 (6.5) | 2460 (9.7) | 0.73 (0.67-0.78, p<0.001) | * |

*Multivariable not reported due to effect modification with omicron

Table S4: Descriptive analysis of non-household contacts and their cases, and results of non-household model

| Non-household | | | | | |
| --- | --- | --- | --- | --- | --- |
|  |  | Delta | Omicron | OR (univariable) | OR (multivariable) |
| Variant | Delta | 22811 (60.7) |  | - | - |
|  | Omicron |  | 14790 (39.3) | 2.30 (2.10-2.52, p<0.001) | * |
| Exposer Age | 0 - 9 | 3032 (13.3) | 435 (2.9) | 0.47 (0.38-0.59, p<0.001) | 0.69 (0.52-0.91, p=0.009) |
|  | 10 - 19 | 3616 (15.9) | 1125 (7.6) | 0.45 (0.37-0.55, p<0.001) | 0.62 (0.49-0.79, p<0.001) |
|  | 20 - 29 | 2362 (10.4) | 4746 (32.1) | 1.38 (1.22-1.57, p<0.001) | 1.17 (1.02-1.34, p=0.023) |
|  | 30 - 39 | 4306 (18.9) | 3814 (25.8) | - | - |
|  | 40 - 49 | 4982 (21.8) | 2532 (17.1) | 0.96 (0.84-1.10, p=0.583) | 1.15 (1.00-1.32, p=0.054) |
|  | 50 - 59 | 3242 (14.2) | 1470 (9.9) | 0.85 (0.72-0.99, p=0.040) | 1.12 (0.95-1.33, p=0.175) |
|  | 60 - 69 | 946 (4.1) | 532 (3.6) | 0.79 (0.61-1.02, p=0.069) | 1.10 (0.84-1.44, p=0.495) |
|  | 70 - 79 | 265 (1.2) | 111 (0.8) | 0.43 (0.23-0.81, p=0.009) | 0.73 (0.38-1.42, p=0.360) |
|  | 80+ | 60 (0.3) | 25 (0.2) | 0.57 (0.18-1.82, p=0.344) | 0.96 (0.29-3.10, p=0.941) |
| Exposer Sex | Female | 13140 (57.6) | 8771 (59.3) | - | - |
|  | Male | 9671 (42.4) | 6019 (40.7) | 0.98 (0.90-1.08, p=0.719) | 1.02 (0.93-1.12, p=0.655) |
| Exposer Ethnicity | Asian | 724 (3.2) | 643 (4.3) | 1.42 (1.15-1.75, p=0.001) | 1.25 (1.00-1.55, p=0.045) |
|  | Black | 206 (0.9) | 881 (6.0) | 1.68 (1.35-2.09, p<0.001) | 1.15 (0.91-1.46, p=0.249) |
|  | Mixed | 511 (2.2) | 704 (4.8) | 1.30 (1.04-1.64, p=0.024) | 1.13 (0.89-1.44, p=0.306) |
|  | Other/Unknown | 983 (4.3) | 798 (5.4) | 1.03 (0.83-1.27, p=0.815) | 0.98 (0.79-1.23, p=0.872) |
|  | White | 20387 (89.4) | 11764 (79.5) | - | - |
| Exposer Region | East Midlands | 1880 (8.2) | 929 (6.3) | - | - |
|  | East Of England | 2681 (11.8) | 1887 (12.8) | 0.85 (0.69-1.06, p=0.159) | 0.80 (0.64-1.00, p=0.053) |
|  | London | 2156 (9.5) | 5129 (34.7) | 1.72 (1.43-2.07, p<0.001) | 1.24 (1.02-1.52, p=0.031) |
|  | Missing | 101 (0.4) | 85 (0.6) | 1.52 (0.86-2.68, p=0.152) | 1.34 (0.73-2.44, p=0.343) |
|  | North East | 1018 (4.5) | 224 (1.5) | 0.93 (0.68-1.27, p=0.646) | 1.07 (0.78-1.46, p=0.692) |
|  | North West | 1785 (7.8) | 1651 (11.2) | 1.05 (0.84-1.31, p=0.678) | 0.97 (0.77-1.22, p=0.813) |
|  | South East | 6468 (28.4) | 2900 (19.6) | 1.00 (0.82-1.21, p=0.966) | 1.00 (0.83-1.22, p=0.961) |
|  | South West | 4147 (18.2) | 509 (3.4) | 0.72 (0.57-0.90, p=0.004) | 0.92 (0.73-1.16, p=0.470) |
|  | West Midlands | 1415 (6.2) | 969 (6.6) | 1.02 (0.80-1.31, p=0.857) | 1.12 (0.87-1.44, p=0.369) |
|  | Yorkshire And Humber | 1160 (5.1) | 507 (3.4) | 0.92 (0.69-1.21, p=0.539) | 0.94 (0.71-1.26, p=0.691) |
| Exposer IMD | 1 - most deprived | 2272 (10.0) | 1757 (11.9) | 0.99 (0.83-1.17, p=0.884) | 0.93 (0.78-1.11, p=0.432) |
|  | 2 | 3772 (16.5) | 2555 (17.3) | 1.21 (1.05-1.40, p=0.009) | 1.11 (0.96-1.28, p=0.169) |
|  | 3 | 4803 (21.1) | 3041 (20.6) | - | - |
|  | 4 | 5110 (22.4) | 3443 (23.3) | 1.11 (0.97-1.27, p=0.142) | 1.12 (0.98-1.29, p=0.099) |
|  | 5 - least deprived | 6845 (30.0) | 3986 (27.0) | 1.04 (0.91-1.18, p=0.574) | 1.13 (0.99-1.30, p=0.080) |
| Exposer Vaccination | Unlinked | 689 (3.0) | 643 (4.3) | 1.02 (0.81-1.28, p=0.856) | * |
|  | Unvaccinated | 6483 (28.4) | 1579 (10.7) | 0.63 (0.55-0.71, p<0.001) | * |
|  | 1 Dose + 21 Days | 1189 (5.2) | 617 (4.2) | 0.64 (0.50-0.81, p<0.001) | * |
|  | 2 Doses + 14 Days | 13248 (58.1) | 10522 (71.1) | - | * |
|  | 3 Doses + 14 Days | 1202 (5.3) | 1429 (9.7) | 0.86 (0.72-1.03, p=0.108) | * |
| Exposer Had Symptoms | True | 21322 (93.5) | 13989 (94.6) | - | - |
|  | False | 1489 (6.5) | 801 (5.4) | 0.69 (0.56-0.86, p=0.001) | 0.89 (0.71-1.11, p=0.298) |
| Date of Exposure | 2021-12-03 | 3295 (14.4) | 580 (3.9) | 0.90 (0.75-1.09, p=0.291) | 1.33 (1.10-1.61, p=0.004) |
|  | 2021-12-04 | 4189 (18.4) | 1540 (10.4) | 1.09 (0.93-1.28, p=0.275) | 1.43 (1.21-1.69, p<0.001) |
|  | 2021-12-05 | 3623 (15.9) | 1440 (9.7) | 0.92 (0.77-1.09, p=0.316) | 1.20 (1.01-1.44, p=0.040) |
|  | 2021-12-06 | 2722 (11.9) | 1528 (10.3) | 0.94 (0.79-1.13, p=0.508) | 1.10 (0.92-1.33, p=0.284) |
|  | 2021-12-07 | 2668 (11.7) | 2547 (17.2) | 0.81 (0.68-0.97, p=0.022) | 0.85 (0.71-1.01, p=0.068) |
|  | 2021-12-08 | 2527 (11.1) | 2465 (16.7) | - | - |
|  | 2021-12-09 | 2035 (8.9) | 2675 (18.1) | 0.97 (0.82-1.16, p=0.757) | 0.94 (0.79-1.12, p=0.466) |
|  | 2021-12-10 | 1451 (6.4) | 1757 (11.9) | 1.04 (0.86-1.25, p=0.715) | 1.04 (0.86-1.26, p=0.700) |
|  | 2021-12-11 | 236 (1.0) | 204 (1.4) | 0.78 (0.49-1.25, p=0.303) | 0.91 (0.57-1.47, p=0.711) |
|  | 2021-12-12 | 65 (0.3) | 54 (0.4) | 0.28 (0.07-1.14, p=0.076) | 0.33 (0.08-1.34, p=0.120) |
| Contact Completed | True | 9817 (43.0) | 6797 (46.0) | - | - |
|  | False | 12994 (57.0) | 7993 (54.0) | 0.43 (0.39-0.47, p<0.001) | 0.87 (0.73-1.05, p=0.147) |
| Contact Age | 0 - 9 | 73 (0.3) | 23 (0.2) | 0.79 (0.36-1.72, p=0.555) | 1.19 (0.53-2.69, p=0.674) |
|  | 10 - 19 | 207 (0.9) | 264 (1.8) | 1.04 (0.75-1.44, p=0.827) | 1.22 (0.86-1.72, p=0.262) |
|  | 20 - 29 | 1620 (7.1) | 2166 (14.6) | 1.23 (1.06-1.42, p=0.006) | 1.07 (0.92-1.25, p=0.368) |
|  | 30 - 39 | 2380 (10.4) | 1891 (12.8) | - | - |
|  | 40 - 49 | 2319 (10.2) | 1342 (9.1) | 0.90 (0.76-1.05, p=0.169) | 0.96 (0.82-1.13, p=0.623) |
|  | 50 - 59 | 1960 (8.6) | 1080 (7.3) | 0.65 (0.54-0.78, p<0.001) | 0.75 (0.62-0.91, p=0.003) |
|  | 60 - 69 | 1402 (6.1) | 631 (4.3) | 0.43 (0.34-0.55, p<0.001) | 0.61 (0.47-0.79, p<0.001) |
|  | 70 - 79 | 694 (3.0) | 216 (1.5) | 0.39 (0.27-0.56, p<0.001) | 0.73 (0.49-1.08, p=0.113) |
|  | 80+ | 118 (0.5) | 26 (0.2) | 0.36 (0.15-0.89, p=0.027) | 0.69 (0.27-1.74, p=0.431) |
|  | Missing | 12038 (52.8) | 7151 (48.4) | 0.33 (0.29-0.38, p<0.001) | 0.87 (0.11-6.89, p=0.892) |
| Contact Sex | Female | 6552 (28.7) | 4281 (28.9) | - | - |
|  | Male | 4202 (18.4) | 3341 (22.6) | 1.07 (0.96-1.19, p=0.222) | 1.02 (0.91-1.14, p=0.716) |
|  | Missing | 12044 (52.8) | 7158 (48.4) | 0.39 (0.35-0.44, p<0.001) | 0.87 (0.11-6.87, p=0.895) |
|  | Not Specified | 13 (0.1) | 10 (0.1) | 1.80 (0.53-6.06, p=0.344) | 1.67 (0.48-5.78, p=0.418) |
| Contact Vaccination | Unlinked | 12830 (56.2) | 7775 (52.6) | 0.34 (0.30-0.37, p<0.001) | * |
|  | Unvaccinated | 545 (2.4) | 448 (3.0) | 1.10 (0.89-1.37, p=0.384) | * |
|  | 1 Dose + 21 Days | 217 (1.0) | 171 (1.2) | 0.91 (0.64-1.31, p=0.626) | * |
|  | 2 Doses + 14 Days | 6590 (28.9) | 4998 (33.8) | - | * |
|  | 3 Doses + 14 Days | 2629 (11.5) | 1398 (9.5) | 0.46 (0.39-0.54, p<0.001) | * |

*Multivariable not reported due to effect modification with omicron
